# Post-Recurrence Treatment Patterns and Outcomes After Curative-Intent Multimodality Strategy in Patients with Pleural Mesothelioma: A Retrospective Cohort Study

**DOI:** 10.3390/jcm15208137

**Published:** 2026-10-20

**Authors:** Oliver Illini, Hannah Fabikan, Irene Berghammer, Julie Krainer-Jacobs, Amelie Schneeweiss-Worel, Leyla Ay, Daniel Moser, Christoph Weinlinger, Anna Lang-Stöberl, Maximilian J. Hochmair, Michal Benej, Tibor Krajc, Thomas Klikovits, Arschang Valipour, Stefan Watzka

**Affiliations:** 1 Department of Respiratory and Critical Care Medicine, Klinik Floridsdorf, Vienna Healthcare Group, 1210 Vienna, Austria; leyla.ay@gesundheitsverbund.at (L.A.); daniel.moser@gesundheitsverbund.at (D.M.); maximilian.hochmair@gesundheitsverbund.at (M.J.H.); arschang.valipour@gesundheitsverbund.at (A.V.); 2 Karl Landsteiner Institute of Lung Research and Pulmonary Oncology, 1210 Vienna, Austria; hannah.fabikan@extern.gesundheitsverbund.at (H.F.); julie.krainer@extern.gesundheitsverbund.at (J.K.-J.); christoph.weinlinger@extern.gesundheitsverbund.at (C.W.); anna.lang-stoeberl@extern.gesundheitsverbund.at (A.L.-S.); 3 Department of Respiratory and Pulmonary Diseases, Klinik Hietzing, Vienna Healthcare Group, 1130 Vienna, Austria; irene.berghammer@gesundheitsverbund.at; 4 Department of Thoracic Surgery, Klinik Floridsdorf, Vienna Healthcare Group, 1210 Vienna, Austriamichal.benej@gesundheitsverbund.at (M.B.); tibor.krajc@gesundheitsverbund.at (T.K.); thomas.klikovits@gesundheitsverbund.at (T.K.); stefan.watzka@gesundheitsverbund.at (S.W.); 5 Karl Landsteiner Institute for Thoracic Surgical Research, Klinik Floridsdorf, 1210 Vienna, Austria; 6 Paracelsus Medical University, 5020 Salzburg, Austria

**Keywords:** pleural mesothelioma, recurrence treatment, lung-sparing surgery, disease recurrence, multimodality therapy

## Abstract

**Background**: Disease recurrence is common after surgery for pleural mesothelioma (PM), and the evidence guiding post-recurrence management remains limited. **Methods**: We conducted a retrospective, single-center cohort study including patients with PM treated within a curative-intent multimodality treatment pathway including surgery aiming for a macroscopic complete resection when feasible between 2001 and 2024. Post-recurrence treatments were categorized as best supportive care only (BSC), systemic therapy alone, or local with or without systemic therapy. Survival and disease-free outcomes were analyzed in relation to treatment patterns and clinical prognostic factors. **Results**: Among 67 patients with a median follow-up time of 107 months from surgery, 58 (86.6%) developed disease recurrence. At first recurrence, 21 patients (36%) received local with or without systemic therapy, 26 (45%) systemic therapy alone, and 11 (19%) BSC. The median post-recurrence survival was 21.7 months for patients receiving local with or without systemic therapy, 10.8 months for systemic therapy alone, and 1.6 months for BSC. Lung-sparing surgery and a disease-free interval of at least 12 months were associated with improved post-recurrence survival and post-recurrence progression-free survival. **Conclusions**: In this real-world cohort of patients with PM and extended follow-up, active post-recurrence treatment was associated with longer post-recurrence survival in selected patients. However, conclusions regarding their comparative effectiveness are limited by the retrospective design, incomplete recurrence-related data, and patient selection at recurrence.

## 1. Introduction

Pleural mesothelioma (PM) is an aggressive malignancy, primarily associated with asbestos exposure. Although the use of asbestos has been heavily restricted in many countries, PM remains a significant public health concern due to its long latency period and the absence of a global asbestos ban [1].

Multimodality therapy consisting of surgery, chemotherapy, and, more recently, immunotherapy is considered the standard of care for selected patients with clinically resectable PM after multidisciplinary evaluation. Among surgical options, lung-sparing approaches (LSS) such as pleurectomy/decortication and extended pleurectomy/decortication have been associated with an improved quality of life and potentially prolonged survival compared to the radical extrapleural pneumonectomy (EPP) [2]. However, the MARS2 trial has introduced uncertainty regarding the role of surgery within multimodality treatment for PM, reporting differences in survival and quality-of-life outcomes between surgery-based and non-surgical approaches [3,4].

Overall, the prognosis for patients with PM remains poor, with a median overall survival (OS) ranging from 9 to 17 months, even following aggressive treatment [5,6,7]. A key factor limiting long-term survival is the high recurrence rate, reported in up to 75% despite curative multimodality therapy [8]. The high recurrence rate poses a challenge for clinicians, as there is no consensus on post-recurrence treatment and management in PM.

Recognizing the lack of standardized approaches for recurrent pleural mesothelioma, we conducted a retrospective cohort analysis of patients previously treated with surgery at a specialized institution, focusing on long-term survival and post-recurrence therapeutic strategies. We aimed to identify clinical factors and therapeutic approaches associated with improved outcomes following disease recurrence.

## 2. Materials and Methods

### 2.1. Study Design

This study is a retrospective, non-interventional, single-center real-world analysis. Patients included in this analysis were diagnosed with PM and treated within a curative-intent multimodality treatment pathway including surgery. The study was approved by the institutional review board of the city of Vienna (EK_14_030_VK). The requirement for informed consent was waived for this retrospective analysis. The study was conducted according to the principles of the Declaration of Helsinki [9].

### 2.2. Patients and Data

We reviewed electronic medical records of all patients with PM treated at a referral center in Vienna, Austria, between 2001 and 2024; data cutoff was February 2025. Patients were eligible if they underwent surgery as part of a curative-intent multimodality treatment pathway. We excluded patients managed without surgery and those undergoing surgery with palliative intent. Tumor stage was reported according to pathological staging. Clinical TNM stage was not systematically available; therefore, the timing and extent of possible preoperative suspicion of stage IV disease could not be reconstructed.

### 2.3. Treatment

Treatment decisions were made within multidisciplinary institutional practice and were generally aligned with contemporaneous guideline-based standards applicable during the study period [10]. In general, curative-intent treatment was based on histological confirmation of PM, usually obtained by pleural biopsy, and multidisciplinary evaluation. The treatment modalities following initial diagnosis were divided into three categories: (1) surgical intervention only, (2) surgical intervention and chemotherapy, and (3) surgical intervention, chemotherapy, and radiotherapy. The surgery-only subgroup consisted of two patients in whom additional therapy could not be delivered before death without evidence of disease progression. These patients were retained in the curative-intent cohort because the initial treatment strategy was curative-intent surgical management, but they did not enter the post-recurrence analyses.

In selected cases, preoperative pleural biopsy was inconclusive or the preoperative working diagnosis was another pleural disease, particularly pleural empyema. The two patients with sarcomatoid histology represented cases in which PM was diagnosed unexpectedly following surgery, with the histological subtype established on final pathological examination. In these patients, PM was diagnosed only after surgical histopathological assessment, and systemic therapy was therefore delivered postoperatively as adjuvant treatment when clinically feasible. In patients with a preoperative diagnosis or strong clinical suspicion of PM, systemic therapy was administered as induction/neoadjuvant treatment before surgery. Surgery was performed with the goal of macroscopic complete resection when feasible. Talc pleurodesis was not routinely performed before planned curative-intent surgery. Its use was individualized, primarily for symptomatic pleural effusions in patients who were not considered candidates for definitive surgical treatment.

Two surgical approaches were distinguished: extrapleural pneumonectomy (EPP) and lung-sparing surgery (LSS; pleurectomy/decortication). Diagnostic and surgical approaches were individualized based on clinical presentation and disease extent, with LSS preferred whenever feasible. EPP was reserved for cases with extensive involvement of both the pleura and lung parenchyma. Surgical candidacy was determined through multidisciplinary assessment, considering technical resectability, performance status, cardiopulmonary reserve, comorbidities, histological findings, and overall oncological suitability. Lymph node involvement was evaluated in the context of disease extent and other prognostic factors rather than as an isolated exclusion criterion. Poor functional status or extensive disease precluding meaningful macroscopic cytoreduction represented important limitations to surgical treatment. Hyperthermic intrathoracic chemotherapy was introduced into institutional practice during the later part of the study period and was administered to only a small number of patients in this cohort. Given the limited number of exposed patients and the long observation period, outcomes were not analyzed separately according to its use.

Adjuvant radiotherapy consisted of hemithoracic pleural and mediastinal radiotherapy with locoregional consolidation intent and was delivered as intensity-modulated radiotherapy at a dose of 54–60 Gy in 2 Gy daily fractions. Radiotherapy was administered when disease distribution and postoperative anatomy permitted treatment of the ipsilateral pleural and mediastinal regions without excessive expected toxicity to the preserved lung. Patients who did not respond to neoadjuvant chemotherapy or were deemed unsuitable for surgery received subsequent radiotherapy when feasible, followed by surveillance or best supportive care only (BSC).

### 2.4. Follow-Up and Recurrence Assessment

After completion of curative-intent treatment, patients underwent routine postoperative surveillance according to institutional practice. Follow-up generally included clinical assessment and contrast-enhanced CT imaging at approximately 3-month intervals during the early postoperative period. During longer-term follow-up, intervals were extended, commonly to 6–9 months, depending on clinical course, prior findings, and physician assessment. PET-CT was not used as a fixed alternating surveillance modality but was performed when recurrence was suspected or when CT findings required further clarification. Recurrence was primarily diagnosed radiologically based on cross-sectional imaging. Biopsy confirmation was obtained when imaging findings were equivocal or when histological confirmation was considered necessary for treatment decision-making.

Post-recurrence treatment was categorized as BSC, local therapy with or without systemic therapy (surgery and/or radiotherapy, alone or combined with systemic therapy), or systemic therapy only.

At recurrence, treatment decisions were made within multidisciplinary institutional practice rather than according to a fixed allocation protocol. Selection of local therapy, systemic therapy, or BSC was individualized based on performance status, comorbidity, pulmonary reserve, recurrence extent and localization, prior treatment exposure, disease-free interval, feasibility of local treatment, expected treatment tolerance, patient preference, and availability of systemic treatment options during the respective treatment era.

### 2.5. Statistical Analysis of Study Endpoints

Overall survival (OS) was defined as the interval from the date of surgery to the date of death or last follow-up. Disease-free survival (DFS) was defined as the interval from surgery to first disease progression or death. Post-recurrence progression-free survival (prPFS) was defined as the interval from first to second disease progression or death. Post-recurrence survival (PRS) was defined as the interval from first disease progression to death or last follow-up. Median follow-up from surgery and from first recurrence was estimated using the reverse Kaplan–Meier method. DFS grouping was not included as a covariate in OS models calculated from surgery to avoid immortal time bias. Post-recurrence treatment was analyzed using post-recurrence endpoints, because treatment at recurrence is not a baseline exposure.

Survival was estimated using the Kaplan–Meier method. Subgroups were compared using the log-rank test. Exploratory analyses were performed using Cox proportional hazards regression. Parameters included age, sex, histological subtype, laterality, treatment modality, Charlson comorbidity index (CCI), type of surgery, disease stage, and DFS group (less than 12 months vs. 12 months or more). Variables that were significant in univariable analyses were included in multivariable models. To explore potential temporal bias, calendar year was evaluated in exploratory analyses. Calendar year of surgery was used for OS and DFS, whereas calendar year of first recurrence was used for PRS and prPFS analyses. Treatment era was additionally evaluated using a dichotomized cutoff (pre-2021 vs. ≥2021). To assess temporal changes in surgical practice, logistic regression was used to evaluate the association between calendar year and surgical approach (LSS vs. EPP). A multivariable Cox model including calendar year of surgery and surgical approach was used to evaluate whether the association between surgical approach and OS was attenuated after adjustment for calendar time.

A *p*-value below 0.05 was considered statistically significant. No adjustment for multiple testing was applied, and *p*-values should be interpreted as exploratory. Continuous variables are reported as mean (SD) or median (IQR), as appropriate. Categorical variables are reported as counts (percentages) and were compared using the chi-square test. All analyses were performed using R (version 4.4.1) [11].

To provide clinical context for the curative-intent cohort, we performed an exploratory post hoc comparator analysis in the overall institutional cohort. OS was calculated from initial diagnosis to death or last follow-up, because surgery-based time origins were not applicable to patients managed without surgery. Patients were grouped by initial treatment strategy: curative-intent surgery-based pathway, management without surgery, or palliative-intent surgery. This analysis was interpreted descriptively because treatment allocation was non-randomized.

## 3. Results

### 3.1. Patient and Treatment Characteristics

**Figure 1:**
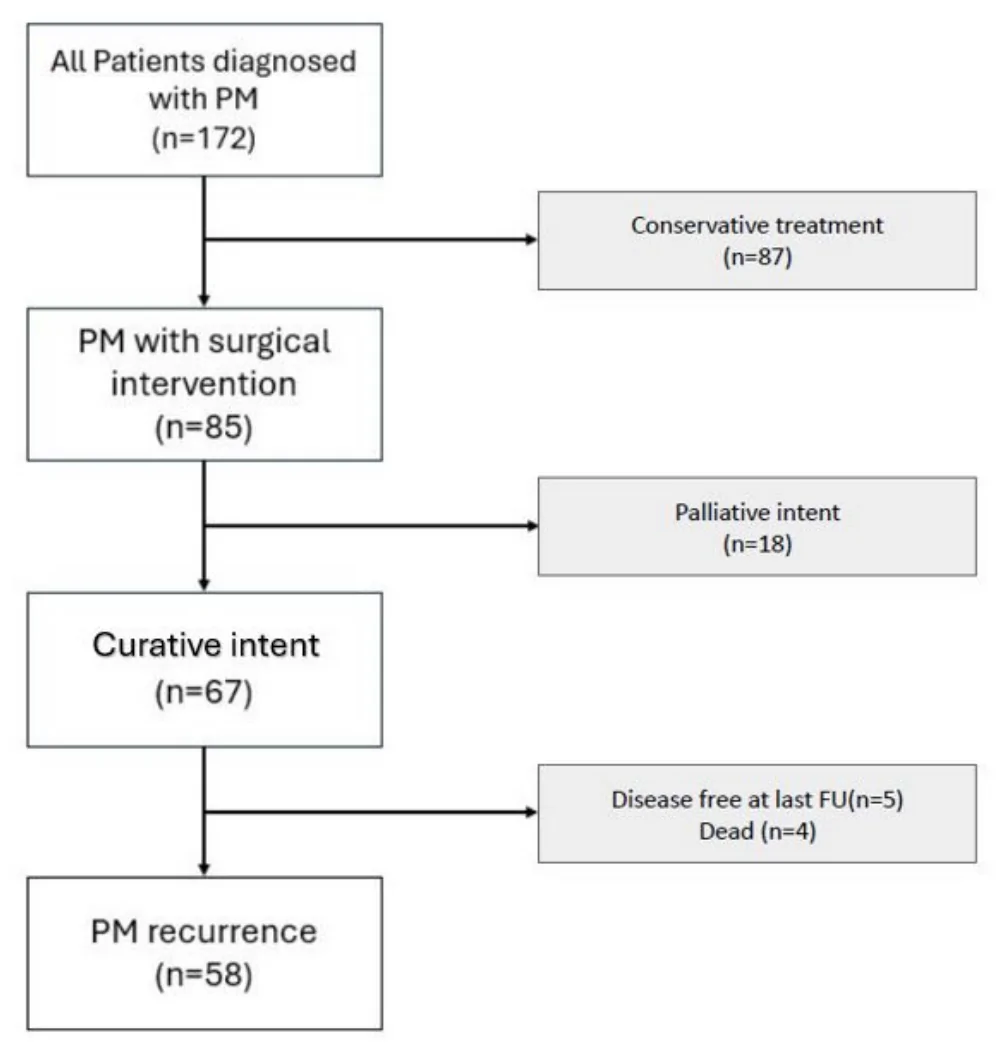
Study population. Flowchart illustrating the distribution of all patients diagnosed with pleural mesothelioma (PM), including those receiving conservative treatment, undergoing surgical intervention with curative or palliative intent, and the number of patients experiencing PM recurrence. Of the nine patients who underwent surgical intervention with curative intent and were excluded from post-recurrence analyses, five were alive and disease-free at data cut-off (last FU), and four died without evidence of disease progression.

Of the 172 patients with PM treated at our institution, 87 were excluded because they did not undergo surgery and 18 were excluded because surgery was performed with palliative intent. The final cohort included 67 patients treated within a curative-intent multimodality treatment pathway including surgery (Figure 1). Among the 85 patients who underwent surgery in the overall institutional cohort, 66 (77.6%) achieved macroscopic complete resection (MCR). MCR status was not used as an exclusion criterion. Among the 67 patients in the curative-intent surgical cohort, postoperative complications occurred in 38 patients (56.7%; Supplementary Table S1). The 30-day and 90-day postoperative mortality rates were 1.5% (1/67) and 3.0% (2/67), respectively. By the data cutoff, 58 patients had developed recurrence and were included in post-recurrence analyses. Of the remaining 9 patients, 5 were alive without progression and 4 had died without evidence of disease progression.

**Figure 2:**
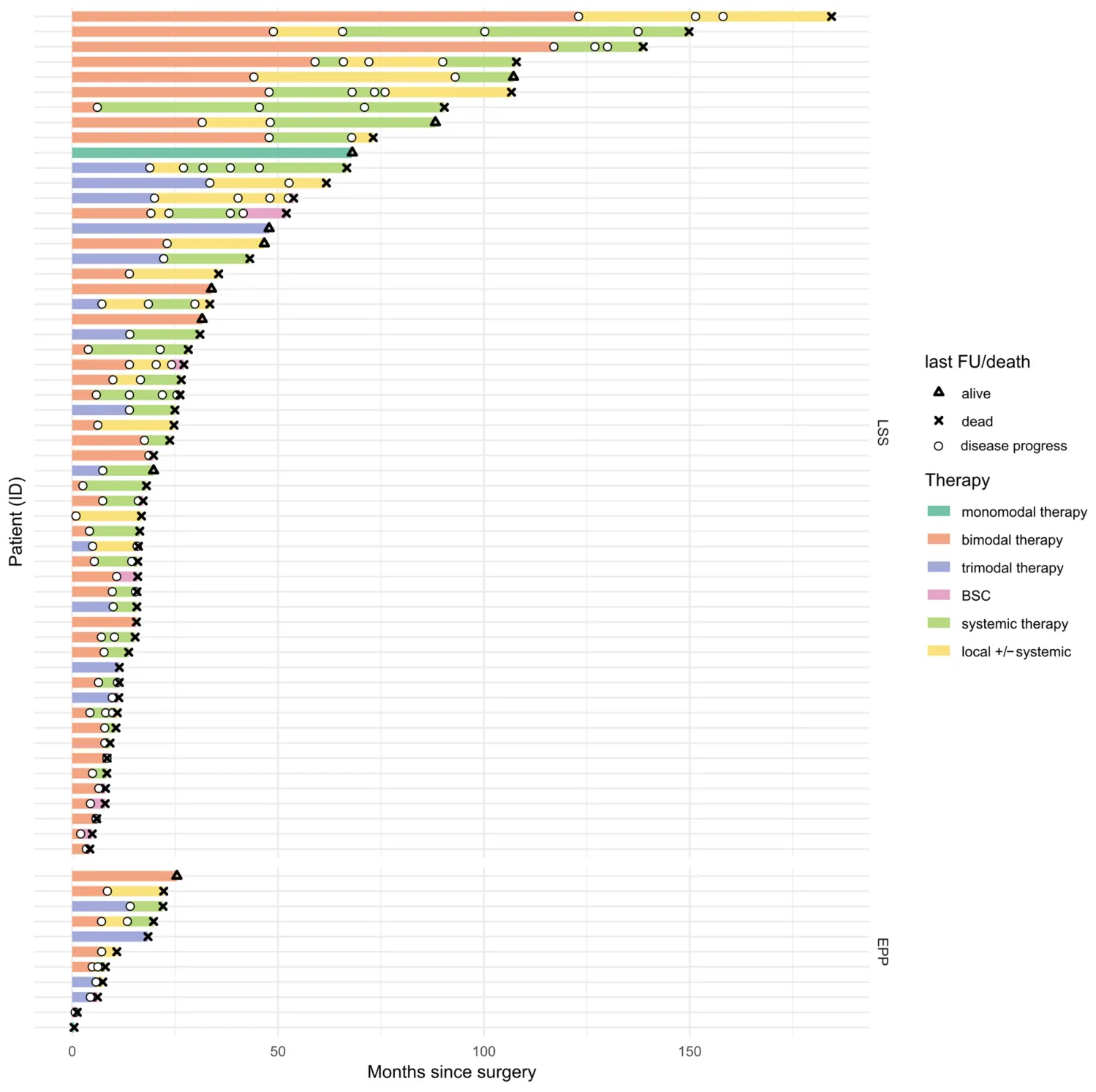
Individual treatment trajectories of all patients, including initial multimodality treatment approaches and therapies administered at recurrence. The different colors represent treatment modalities (monomodal, bimodal, trimodal, systemic, local +/− systemic, or best supportive care), while symbols indicate disease progression, last follow-up, or death.

Patient characteristics (mean age 62 years, 30% female) are summarized in Table 1. The predominant histological subtype was epithelioid (89.6%). Figure 2 illustrates the treatment trajectories of individual patients, including the initial treatment approach and therapies administered at recurrence. At diagnosis, most patients received bi- or trimodality therapy. LSS was performed in 56 patients (83.6%), and the remaining 11 patients (16.4%) underwent EPP. At data cutoff, 58 patients (86.6%) experienced PM recurrence.

In an exploratory post hoc comparator analysis of the overall institutional cohort (*n* = 172), the OS from initial diagnosis differed by initial treatment strategy. Patients treated within the curative-intent surgery-based pathway (*n* = 67) had a median OS of 28.2 months (95% CI, 21.23–35.6), compared with 11.1 months (95% CI, 8.52–14.6) in patients managed without surgery (*n* = 87). Patients undergoing palliative-intent surgery (*n* = 18), who were described separately, had a median OS of 17.4 months (95% CI, 7.84–34.9). In the overall three-group comparison, the OS differed significantly by initial treatment strategy (log-rank *p* < 0.001).

### 3.2. Overall Survival and Disease-Free Survival

**Figure 3:**
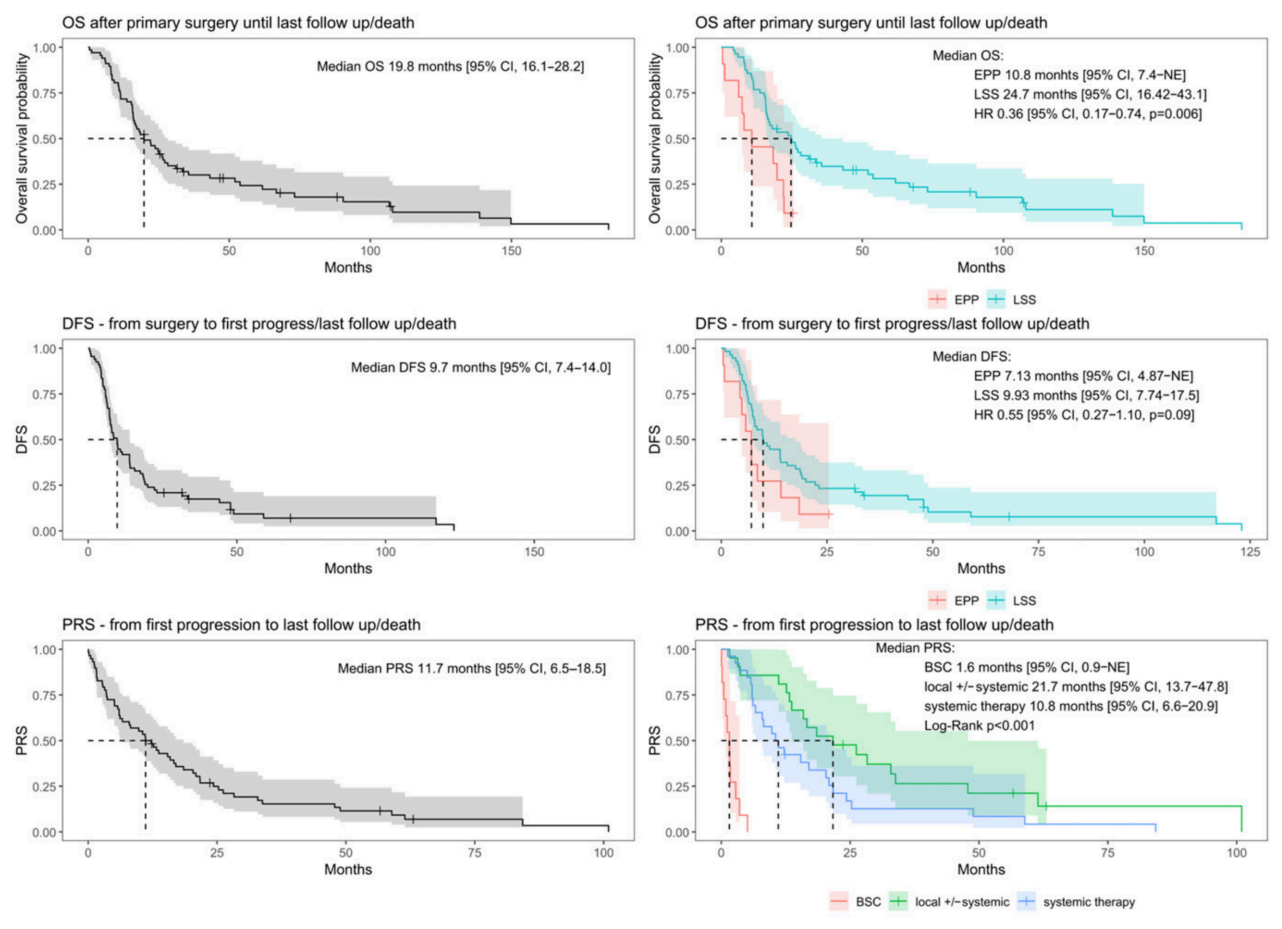
Kaplan–Meier curves showing overall survival (OS) and disease-free survival (DFS) for the entire cohort (**left**) and stratified by extrapleural pneumonectomy (EPP) and lung-sparing surgery (LSS) (**right**) from primary surgery, and post-recurrence survival (PRS) from first progression for the entire cohort (**left**) and stratified by treatment regimen (**right**). Median survival times with 95% confidence intervals and hazard ratios are indicated where applicable. Shaded areas represent 95% confidence bands. Dashed lines indicate the median survival estimates.

**Figure 4:**
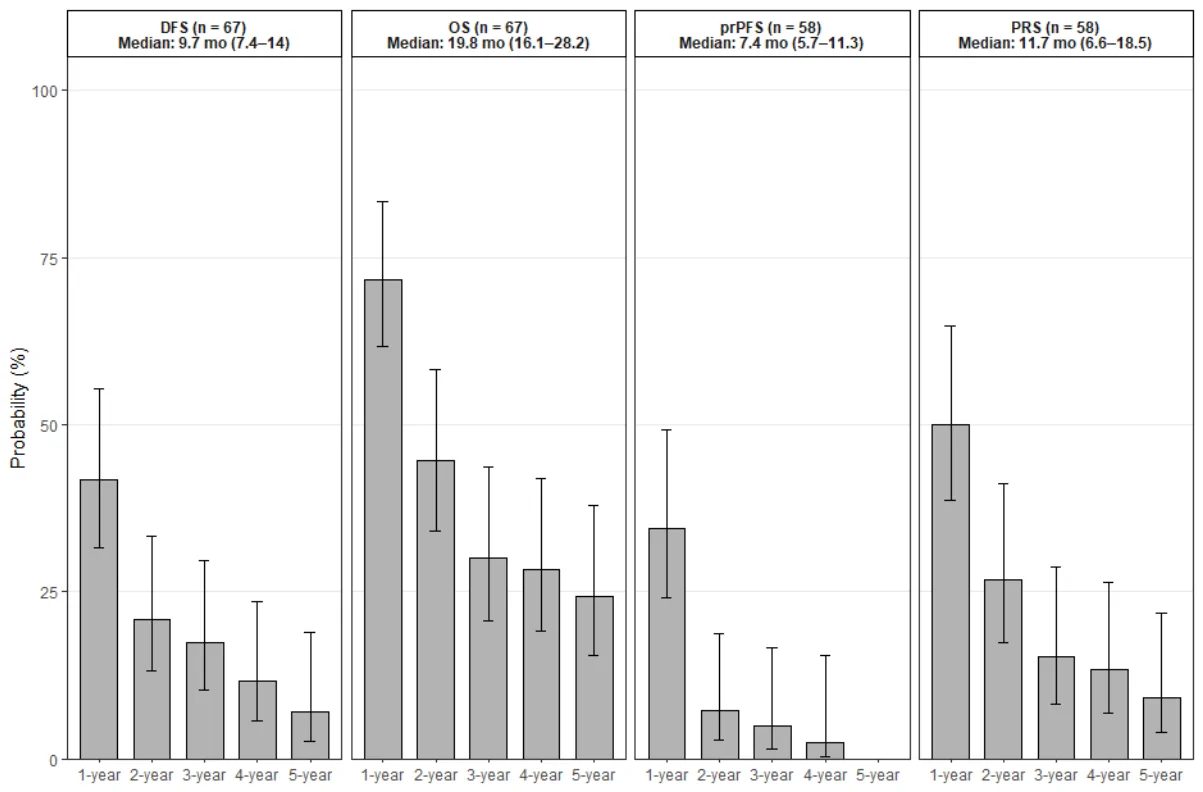
Time-point estimates for DFS, OS, prPFS, and PRS. Bars show Kaplan–Meier estimated probabilities at predefined time points. Error bars indicate 95% confidence intervals. For prPFS, the 5-year estimate was not estimable and is therefore not displayed. DFS, disease-free survival; OS, overall survival; prPFS, post-recurrence progression-free survival; PRS, post-recurrence survival; CI, confidence interval; mo, months.

The median follow-up from surgery was 107 months (95% CI, 68-not estimable). The median OS after surgery was 19.8 months (95% CI, 16.1–28.2) (Figure 3). The 1-year, 2-year, and 5-year OS rates were 72%, 45%, and 24%, respectively. Time-point estimates for OS, DFS, PRS, and prPFS are summarized graphically in Figure 4, with detailed numerical estimates provided in Supplementary Table S2.

In a univariable analysis of prognostic factors, sex and type of surgery were significantly associated with OS. Table 2 summarizes the prognostic factor analyses reported for the study endpoints. Patients undergoing LSS had a longer median OS (25 months) compared with those undergoing EPP (11 months; HR 0.36, 95% CI 0.17–0.74, *p* = 0.006). In a multivariable analysis, LSS remained significantly associated with a longer OS.

The median DFS was 9.7 months (95% CI, 7.4–14.0). The 1-year, 2-year, and 5-year DFS rates were 42%, 21%, and 7%, respectively. In univariable analysis, the CCI group was significantly associated with DFS; patients with CCI 0–5 had a longer DFS than those with CCI > 5 (HR 0.55, 95% CI 0.31–0.97, *p* = 0.041).

### 3.3. Post-Recurrence Survival (PRS) and Post-Recurrence Progression-Free Survival (prPFS)

Of the 58 patients who experienced disease recurrence, 21 (36%) received local +/− systemic therapy, 26 (45%) received systemic therapy alone, and 11 (19%) received BSC. Among these patients, the median potential follow-up from first recurrence was 63.1 months (95% CI, 63.0-not estimable).

Among the 21 patients receiving local +/− systemic therapy at first recurrence, the treatments were heterogeneous and included radiotherapy alone (*n* = 8), surgery alone (*n* = 5), surgery plus chemotherapy (*n* = 3), radiotherapy plus chemotherapy (*n* = 3), trimodality treatment with surgery, chemotherapy, and radiotherapy (*n* = 1), and multimodality treatment including surgery, radiotherapy, chemotherapy, and immunotherapy (*n* = 1). Surgical treatment at first recurrence consisted of selected local resections of circumscribed, technically resectable disease, undertaken with the intent of a complete local excision. Surgery was considered only when a complete local resection appeared feasible; purely palliative surgical procedures were not included in this treatment category. Systemic treatment regimens at first recurrence included carboplatin/pemetrexed (*n* = 14), cisplatin/pemetrexed (*n* = 5), pemetrexed alone (*n* = 3), vinorelbine (*n* = 2), nivolumab/ipilimumab (*n* = 4), carboplatin/pemetrexed/bevacizumab (*n* = 1), raltitrexed/oxaliplatin (*n* = 1), and unknown regimens (*n* = 4). Eleven patients experienced first disease progression in or after 2021; among these, four received nivolumab/ipilimumab at first recurrence. The small number of patients treated with nivolumab/ipilimumab precluded robust subgroup analyses or reliable assessment of immunotherapy-specific outcomes. At second recurrence (*n* = 29), 7 patients (24.1%) underwent further local treatment (4 radiotherapy, 3 surgery), 17 (58.6%) received systemic therapy alone, and 5 (17.3%) received BSC.

The median PRS was 11.7 months (95% CI, 6.6–18.5). The median PRS was 1.6 months for patients managed with BSC (95% CI, 0.9-not estimable), 21.7 months for patients receiving local +/− systemic therapy (95% CI, 13.7–47.8), and 10.8 months for patients receiving systemic therapy alone (95% CI, 6.6–20.9). In the multivariable analysis, local +/− systemic therapy was associated with a longer PRS compared with BSC (HR 0.05, 95% CI 0.02–0.15, *p* < 0.001), whereas the comparison with systemic therapy alone showed borderline significance (HR 0.51, 95% CI 0.26–1.00, *p* = 0.051). LSS was associated with a longer PRS compared with EPP (HR 0.28, 95% CI 0.12–0.68, *p* = 0.005).

The 1-year and 2-year prPFS rates were 34% and 7.3%, respectively; the 5-year prPFS estimate was not estimable. Post-recurrence progression-free survival also differed across first-recurrence treatment groups; these estimates are summarized in Table 3. LSS was associated with a longer prPFS than EPP (HR 0.37, 95% CI 0.16–0.84, *p* = 0.018). Patients with DFS ≥ 12 months had a longer PRS (HR 0.40, 95% CI, 0.20–0.78, *p* = 0.007) and longer prPFS (HR 0.51, 95% CI 0.27–0.96, *p* = 0.038). In exploratory analyses of temporal trends, the calendar year of the first recurrence was not significantly associated with PRS (HR 0.96 per year, 95% CI, 0.90–1.01; *p* = 0.126) or prPFS (HR 0.97 per year, 95% CI, 0.92–1.02; *p* = 0.258). In contrast, a later calendar year of surgery was associated with an improved OS from surgery (HR 0.94 per year, 95% CI, 0.89–0.99; *p* = 0.031), whereas only a non-significant trend was observed for DFS (HR 0.95 per year, 95% CI, 0.91–1.00; *p* = 0.061). The surgical approach changed over time, with LSS being more frequently performed in later years (OR 1.21 per year, 95% CI, 1.04–1.47; *p* < 0.05). In multivariable Cox regression including both the calendar year of surgery and surgical approach, the association between the calendar year and OS was attenuated and no longer statistically significant (HR 0.96 per year, 95% CI, 0.91–1.02; *p* = 0.184), whereas LSS versus EPP remained associated with an improved OS (HR 0.38, 95% CI, 0.17–0.84; *p* = 0.017). Stratified analyses by treatment era (pre-2021 vs. ≥2021) did not demonstrate significant differences in survival outcomes.

## 4. Comment

In this retrospective, single-center cohort of pleural mesothelioma patients managed within a curative-intent multimodality treatment pathway that included surgery aiming for macroscopic complete resection when feasible, we analyzed long-term outcomes with a specific focus on post-recurrence management.

Three findings stand out. First, recurrence occurred in most patients despite curative-intent management. Second, active treatment at recurrence (local therapy with or without systemic therapy, or systemic therapy alone) was associated with a longer PRS than BSC only. Third, disease-free interval and clinical context at recurrence appeared to stratify prognosis and may help identify patients most likely to be considered for active post-recurrence treatment. Overall, these observations align with prior institutional series and add real-world data over a long observational period.

In our cohort, 58 of 67 patients (86.6%) developed recurrence. Comorbidity burden, as reflected by the CCI, was associated with DFS in univariable Cox regression, suggesting that baseline patient factors may influence the recurrence risk and timing. However, comorbidity may also affect treatment intensity and selection, which could confound the observed associations in this retrospective cohort. High recurrence rates have been reported after both EPP and LSS, even in selected cohorts [12,13]. In this context, surgery is best viewed as one component of a broader disease-control strategy rather than a standalone definitive curative modality. This framing is also consistent with the practical limitation that surgery in PM generally aims for macroscopic complete resection rather than microscopic clearance.

Recent randomized evidence has challenged the role of surgery in PM, particularly after MARS2, which demonstrated inferior survival and quality-of-life outcomes for systemic therapy plus extended pleurectomy/decortication compared with systemic therapy alone [3,4]. However, MARS2 did not address post-recurrence treatment strategies, nor did it evaluate long-term survivors beyond recurrence. Our analysis therefore focuses on a clinically underexplored phase in the disease course: management after recurrence in patients initially selected for curative-intent treatment within a multimodality strategy.

The exploratory comparator analysis provides clinical context for the selected curative-intent cohort but should not be interpreted as evidence of treatment efficacy. Patients selected for curative-intent surgery differed from those managed without surgery or with palliative-intent surgery with respect to treatment intent, operability, disease extent, functional reserve, baseline prognosis, and treatment era. Therefore, the observed survival differences cannot be attributed to surgery itself, and the relative contributions of patient selection and surgical treatment cannot be determined. This post hoc comparison is descriptive and hypothesis-generating, while the main objective of this study remains post-recurrence management after curative-intent surgery.

The OS observed in our cohort is broadly consistent with real-world surgical series treated within multimodality strategies, acknowledging that cross-study comparisons are limited by differences in patient selection, treatment strategies, and follow-up duration [14,15]. In recurrence-focused cohorts, Bellini et al. and Nakamura et al. reported a longer OS measured from the time of diagnosis, which likely reflects the differences in cohort composition and selection rather than superior treatment efficacy [13,14]. At recurrence, local treatment was not confined to one modality but encompassed heterogeneous surgical and radiotherapeutic approaches, frequently combined with systemic therapy. This heterogeneity reflects real-world decision-making based on the recurrence pattern, functional status, and prior treatment exposure rather than a standardized algorithm.

Post-recurrence survival differed by treatment strategy. The longest survival was observed in patients receiving local therapy with or without systemic therapy, followed by systemic therapy alone, whereas patients managed with BSC had the shortest survival. These differences should not be interpreted as direct treatment effects. Treatment allocation at recurrence was individualized and strongly influenced by performance status, comorbidity, pulmonary reserve, disease burden, recurrence pattern, patient preference, and prior treatment exposure. Because several key determinants of treatment allocation were not available in a standardized analyzable form, meaningful adjustment for treatment-selection bias was not possible. Accordingly, comparisons among local therapy, systemic therapy, and BSC should be interpreted as descriptive outcome patterns rather than evidence of comparative treatment efficacy. Nevertheless, the observed gradient is consistent with recurrence-focused series reporting prolonged survival in selected patients able to undergo active post-recurrence treatment [13,14].

Several factors were associated with longer survival in our cohort, including lung-sparing surgery compared with extrapleural pneumonectomy. The association between epithelioid histology and improved post-recurrence survival in univariable analysis is consistent with prior literature, but this finding did not persist in multivariable analysis and should be interpreted cautiously given the small number of non-epithelioid cases typically selected for curative-intent surgery [15].

The association between LSS and longer survival outcomes must be interpreted cautiously because the surgical approach was confounded by calendar time and patient selection. EPP was performed predominantly earlier in the study period, whereas LSS became more frequent in later years. Although exploratory models suggested that the association between LSS and OS persisted after adjustment for calendar year, this should not be interpreted as evidence of a causal effect. The small number of EPP patients limits robust adjustment and era-stratified comparisons, and residual temporal and selection bias cannot be excluded. A plausible explanation is that lung-sparing procedures may preserve functional reserve, potentially increasing eligibility for additional local or combined post-recurrence treatments, whereas patients after pneumonectomy may have fewer subsequent options. However, this remains a hypothesis and cannot be formally tested in the present dataset.

Anatomical recurrence patterns were not systematically captured in our cohort, limiting the direct comparison with reports emphasizing local versus distant relapse patterns. Bellini et al. reported a higher incidence of distant recurrence after EPP, whereas Nakamura et al. observed a predominance of local recurrence after pleurectomy/decortication [13,14]. In our cohort, a disease-free interval of at least 12 months emerged as one of the strongest prognostic factors and was associated with a longer PRS and prPFS. This is consistent with prior reports, suggesting that longer disease-free intervals may reflect more favorable tumor biology and are associated with improved outcomes after recurrence [12,15]. This variable is also clinically pragmatic and may support stratification in future observational analyses and trial designs.

The interpretation of post-recurrence outcomes is further complicated by the long inclusion period and evolving systemic treatment landscape. Immune checkpoint inhibition changed the standards for unresectable disease and became available only during the later part of the study period [16]. In our cohort, only a small number of patients received nivolumab/ipilimumab at first recurrence, and exploratory treatment-era analyses did not demonstrate significant survival differences between patients recurring before 2021 and those recurring in or after 2021. Therefore, the potential impact of immunotherapy on post-recurrence outcomes could not be reliably assessed. Biomarker-driven strategies and perioperative systemic approaches remain under active investigation, and their role in guiding post-recurrence decisions is not yet established [17,18,19,20,21]. The implementation of emerging therapeutic approaches at recurrence is further complicated by impaired performance status, reduced functional reserve following previous treatment, cumulative toxicity, and limited remaining treatment options. In addition, restricted access to newer or off-label therapies and the limited prospective evidence available for recurrent PM may further constrain treatment decisions. These challenges emphasize the importance of individualized multidisciplinary assessment.

This study is limited by its retrospective design, selection bias in surgical candidacy and post-recurrence treatment allocation, and temporal heterogeneity across the long study period. Furthermore, the clinical TNM stage was not systematically available, and staging analyses were therefore restricted to the pathological stage; consequently, the relatively high proportion of pathological stage IV disease could not be contextualized by the preoperative disease extent. Detailed anatomical recurrence patterns were not systematically available, limiting both comparisons with series that stratify outcomes by relapse distribution and the interpretation of the treatment selection at recurrence. Because the clinical disease extent and relapse distribution likely influenced both treatment allocation and prognosis, the observed differences in PRS and prPFS cannot be attributed to treatment strategy alone and should be regarded as descriptive associations. In addition, the MCR status was not used as an exclusion criterion. Therefore, in patients without MCR, subsequent disease events may represent the progression of residual macroscopic disease rather than true recurrence after a complete macroscopic resection. This limitation affects the interpretation of DFS, disease-free interval, and post-recurrence endpoints, and may limit the comparability with recurrence-focused surgical series reporting higher MCR rates. These limitations also highlight the need for prospective multicenter registries with the standardized documentation of clinical staging, performance status, anatomical recurrence patterns, disease burden, treatment eligibility, and longitudinal outcomes. Such efforts would improve the interpretability of real-world evidence and facilitate more meaningful comparisons of treatment strategies in this rare disease.

Recurrence after initial curative-intent management should not be equated with treatment futility. In this cohort, active post-recurrence treatment was associated with a longer post-recurrence survival, particularly in patients with longer disease-free intervals. However, the observed survival differences between post-recurrence treatment groups should not be interpreted as evidence of comparative treatment efficacy. These findings support structured multidisciplinary reassessment at recurrence.

## Figures and Tables

**Table 1:** Patient characteristics.

Characteristics	All Patients *n* = 67	PM Recurrence *n* = 58
Age, mean (range)	62 (37–81)	62 (37–81)
Sex, *n* (%) female	20 (29.9)	18 (31.0)
Charlson comorbidity index		
CCI mean (IQR)	4.4 (2–6)	4.7 (3–7)
CCI 0–5, *n* (%)	49 (73.1)	40 (69.0)
CCI >5, *n* (%)	18 (26.9)	18 (31.0)
Histology, *n* (%)		
Epithelioid	60 (89.6)	52 (89.7)
Sarcomatoid	2 (3.0)	1 (1.7)
Biphasic	5 (7.5)	5 (8.6)
Side, *n* (%)		
Right	43 (64.2)	38 (65.5)
Left	24 (35.8)	20 (34.5)
Pathological stage (TNM VIII edition), *n* (%)		
Complete remission	1 (1.5)	1 (1.7)
I	18 (26.9)	14 (24.1)
II	10 (14.9)	9 (15.5)
III	24 (35.8)	23 (39.7)
IV	9 (13.4)	9 (15.5)
Unknown	5 (7.5)	2 (3.4)
Treatment, *n* (%)		
Surgical intervention ^c^	2 (3.0)	0 (0.0)
Bimodal ^a^	48 (71.6)	44 (75.9)
Trimodal ^b^	17 (25.4)	14 (24.1)
Surgical intervention, *n* (%)		
EPP, *n* (%)	11 (16.4)	8 (13.8)
LSS, *n* (%)	56 (83.6)	50 (86.2)
DFS, *n* (%)		
<12 months	39 (58.2)	37 (63.8)
≥12 months	28 (41.8)	21 (36.2)
**Post-recurrence treatment**		
Treatment of first recurrence (*n* = 58), *n* (%)		
BSC	11 (19.0)	
Local +/− systemic	21 (36.2)	
Systemic	26 (44.8)	
Treatment of second recurrence (*n* = 29), *n* (%)		
BSC	5 (17.2)	
Local +/− systemic	7 (24.1)	
Systemic	17 (58.6)	

^a^ Bimodal therapy consists of chemotherapy + surgery; ^b^ Trimodal therapy consists of chemotherapy + surgery + radiotherapy; ^c^ Surgery-only cases represent patients selected for a curative-intent multimodality pathway including surgery in whom additional planned therapy could not be delivered before death without evidence of disease progression.

**Table 2:** Prognostic factors for OS, DFS, PRS, and prPFS.

	*n*		*p*-Value	HR	95% CI
OS	67				
		LSS vs. EPP	0.025	0.42	0.20–0.90
DFS	67				
		CCI 0–5 vs. CCI >5	0.041	0.55	0.31–0.97
PRS	58				
		LSS vs. EPP	0.005	0.28	0.12–0.68
		Local +/− systemic vs. systemic therapy	0.051	0.51	0.26–1.00
		Local +/− systemic vs. BSC	<0.001	0.05	0.02–0.15
		DFS ≥ 12 mo	0.007	0.40	0.20–0.78
prPFS	58				
		LSS vs. EPP	0.018	0.37	0.16–0.84
		DFS ≥ 12 mo	0.038	0.51	0.27–0.96
		Local +/− systemic vs. BSC	<0.001	0.07	0.02–0.19

For overall survival (OS), post-recurrence survival (PRS), and post-recurrence progression-free survival (prPFS) multivariate analysis is shown. For disease-free survival (DFS), only univariable analysis is reported because only one covariate was significant in univariable analysis.

**Table 3:** Post-recurrence progression-free survival by treatment group: best supportive care, local therapy with or without systemic therapy, and systemic therapy alone.

Treatment Group	Median prPFS	1-Month Progression-Free Probability (95% CI)	12-Month Progression-Free Probability (95% CI)	24-Month Progression-Free Probability (95% CI)	48-Month Progression-Free Probability (95% CI)
BSC	1.6 (0.89, —)	64% (41%, 99%)	— (—, —)	— (—, —)	— (—, —)
Local +/− systemic	14 (6.7, 20)	100% (100%, 100%)	52% (35%, 79%)	14% (5.0%, 41%)	7.1% (1.3%, 41%)
Systemic	8.3 (6.0, 17)	100% (100%, 100%)	35% (20%, 59%)	4.4% (0.7%, 30%)	— (—, —)

## Data Availability

De-identified data is available upon reasonable request to the corresponding author, subject to institutional approvals.

## Supplementary Materials

The following supporting information can be downloaded at: https://www.mdpi.com/article/10.3390/jcm15208137/s1, Table S1. Postoperative complications in patients undergoing curative-intent surgery for pleural mesothelioma. Table S2. Kaplan-Meier survival estimates at predefined time points for overall survival (OS), disease-free survival (DFS), post-recurrence survival (PRS), and post-recurrence progression-free survival (prPFS), including 95% confidence intervals.
