# Supplementary material for: Post-Recurrence Treatment Patterns and Outcomes After Curative-Intent Multimodality Strategy in Patients with Pleural Mesothelioma: A Retrospective Cohort Study
Source: J Clin Med. 2026 Oct 20;15(20):8137. doi: 10.3390/jcm15208137 (PMC13706363; doi:10.3390/jcm15208137)
Supplement: Supplementary file 1 [file jcm-15-08137-s001.zip › jcm-4570966-supplementary.pdf]

Supplementary Materials

Supplementary Table S1. Postoperative complications in patients undergoing curative-intent surgery for pleural mesothelioma.

**Surgical Complications, n (%)**	**n=67**
Any complications	38 (56.7%)
Postoperative airleak	10 (14.9%)
Atrial fibrillation	9 (13.4%)
Blood transfusion required	8 (11.9%)
Post-pneumonectomy space with mediastinal shift	6 (9.0%)
Failure of the lung to expand	5 (7.5%)
Cardiac decompensation	4 (6.0%)
Renal insufficiency	4 (6.0%)
Postoperative empyema	3 (4.5%)
Pneumonia	3 (4.5%)
Postoperative airleak treated with endobronchial valve placement	3 (4.5%)
Emergency surgery due to bleeding	2 (3.0%)
Subcutaneous emphysema	2 (3.0%)
Wound healing disorder	2 (3.0%)
Hyperglycemia	1 (1.5%)
Perioperative myocardischemia	1 (1.5%)
Pulmonary edema	1 (1.5%)
Gastric herniation	1 (1.5%)
Hyperfibrinolysis	1 (1.5%)
Hypertensive derailment	1 (1.5%)
Postoperative abscess	1 (1.5%)
Right-sided acute cardiac decompensation	1 (1.5%)
Rupture of the diaphragmatic patch	1 (1.5%)
Urinary tract infection	1 (1.5%)
Wound infection	1 (1.5%)

Percentages refer to the proportion of patients experiencing each complication. Categories are not mutually exclusive, as individual patients may have experienced more than one postoperative complication.

Supplementary Table S2. Kaplan-Meier survival estimates at predefined time points for overall survival (OS), disease-free survival (DFS), post-recurrence survival (PRS), and post-recurrence progression-free survival (prPFS), including 95% confidence intervals.

	n	Median (95% CI)	1-year (95% CI)	2-year (95% CI)	3-year (95% CI)	4-year (95% CI)	5-year (95% CI)
OS	67	19.8 (16.1, 28.2)	72% (62%, 83%)	45% (34%, 58%)	30% (21%, 44%)	28% (19%, 42%)	24% (16%, 38%)
DFS	67	9.7 (7.4, 14.0)	42% (32%, 55%)	21% (13%, 33%)	17% (10%, 30%)	12% (5.7%, 23%)	7% (2.6%, 19%)
PRS	58	11.7 (6.6, 18.5)	47% (36%, 61%)	25% (16%, 39%)	14% (7.6%, 27%)	13% (6.4%, 25%)	8.6% (3.6%, 20%)
prPFS	58	7.4 (5.7, 11.3)	34% (24%, 49%)	7.3% (2.8%, 19%)	4.9% (1.4%, 17%)	2.4% (0.4%, 16%)	—
